# A randomised controlled trial of probiotics for the prevention of spontaneous preterm delivery associated with bacterial vaginosis: preliminary results

**DOI:** 10.1186/1745-6215-12-239

**Published:** 2011-11-08

**Authors:** Leticia Krauss-Silva, Maria Elizabeth L Moreira, Mariane B Alves, Alcione Braga, Karla G Camacho, Maria Rosa R Batista, Antonio Almada-Horta, Maria R Rebello, Fernando Guerra

**Affiliations:** 1 Health Technology Assessment Unit, National School of Public Health, Oswaldo Cruz Foundation, Brazilian Health Ministry, Brazil, R. Leopoldo Bulhões, 1480, Rio de Janeiro, 21041-210, Brazil; 2 Clinical Research Unit, Fernandes Figueira Institute, Oswaldo Cruz Foundation, Brazilian Health Ministry, Av. Rui Barbosa, 716, Rio de Janeiro, 22250-020, Brazil; 3 Institute of Mathematics, Federal University in Rio de Janeiro, Av. Athos da Silveira Ramos - 149, Rio de Janeiro, 21941-909, Brazil; 4 PROCEP, Pró-Cardíaco, R. General Polidoro, 142, Rio de Janeiro, 22280-003, Brazil; 5 Department of Obstetrics and Gynecology, Fernandes Figueira Institute, Oswaldo Cruz Foundation, Brazilian Health Ministry, Av. Rui Barbosa, 716, Rio de Janeiro, 22250-020, Brazil; 6 Federal University in Rio de Janeiro Medical School, Av. Brigadeiro Trompowski, Rio de Janeiro, 21044-020, Brazil

**Keywords:** spontaneous preterm delivery, prevention, randomised controlled parallel-group trial, probiotics, history of preterm delivery

## Abstract

**Background:**

Bacterial vaginosis increases the risk of spontaneous preterm delivery at less than 34 weeks of gestation.

**Objective:**

The purpose of this study was to evaluate the efficacy of the early administration of selected lactobacilli strains (probiotics) to pregnant women with asymptomatic bacterial vaginosis/intermediate-degree infections to prevent spontaneous premature delivery and associated neonatal morbidity.

**Methods/Design:**

Asymptomatic pregnant women at less than 20 weeks of gestation, with no indication of elective preterm delivery, with a vaginal pH ≥ 4.5 and Nugent score > 3 were randomly assigned to the placebo or intervention group (oral administration of selected lactobacilli up to the 24th to 26th week of gestation). The randomisation was stratified for the history of premature delivery (HPD) and blocked. The allocation was concealed, and the participating health professionals and patients were blinded. The primary outcome was preterm delivery (<34 to <32 weeks), and the secondary outcomes were associated neonatal complications.

**Results:**

In total, 4,204 pregnant women were screened; 320 and 324 individuals were respectively randomly assigned to the placebo and intervention groups, and 62% finished the trial. None of the randomised patients were lost to follow-up. For the non-HPD stratum, the intent-to-treat relative risks of spontaneous premature birth at < 34 and < 37 weeks' gestation were 0.33 (0.03, 3.16) and 0.49 (0.17, 1.44), respectively, and they were non-significant (ns) with p = 0.31 and 0.14. The corresponding actual treatment figures were zero and 0.32 (0.09, 1.19), which were ns with p = 0.12 and 0.06. The intent-to-treat relative risk of spontaneous premature birth at < 37 weeks of gestation for the trial as a whole, including HPD and non-HPD participants, was 0.69 (0.26, 1.78), p = 0.30 (ns). The neonatal complications under evaluation occurred in only one infant (< 34 weeks; placebo group) who presented with respiratory distress syndrome and suspected early neonatal sepsis. The recorded adverse events were minor and relatively non-specific.

**Conclusions:**

The efficacy of the tested probiotics to prevent preterm delivery among women without a history of preterm delivery was not determined because the study sample was insufficient to estimate statistically significant intent-to-treat effects; additional studies are needed to evaluate this intervention among these women.

**Trial registration:**

Trial registration at NIH register: NCT00303082. Sources of funding: the Brazilian Health Ministry and the State of Rio de Janeiro Research Foundation.

## 1- Background

High rates of preterm delivery, i.e., above 10%, remain prevalent in developing and developed regions around the world; preterm birth is associated with nearly 80% of foetal, neonatal, and infant deaths [1-3].

Approximately 25% of preterm deliveries occur as the result of medical indications, and the remaining cases occur spontaneously [4]. Spontaneous preterm deliveries that occur prior to 35 weeks of gestation, and before the 32^nd ^week in particular, have been strongly associated with intrauterine infections, including bacterial vaginosis (BV) [5,6]. A history of preterm delivery (HPD) is the most important factor in spontaneous preterm delivery; HPD increases the risk of a subsequent preterm delivery by threefold and is likely associated with underlying risk factors [4,7,8]. Important risk factors for spontaneous preterm delivery, such as age < 18 years, race, education, and bacterial vaginosis, are associated with a low socioeconomic status. BV doubles the risk of preterm delivery prior to 35 weeks of gestation [7]. BV is present in 15-20% of normal pregnant women in developed countries [7], and this proportion is almost twice as high in high-risk populations [9].

Prematurity resulting from intrauterine infections increases the probability of complications, including early sepsis, bronchopulmonary dysplasia, periventricular leukomalacia, and necrotising enterocolitis [10-13], which may cause long-term neurological disabilities and death.

Bacterial vaginosis (BV) is a modification of the vaginal flora that is characterised by a diminished or absent flora of lactobacilli, which increases the vaginal pH and leads to a significantly increased colonisation by several anaerobic or facultative microorganisms, including *Gardenerella vaginalis*, *Prevotella *sp., *Bacteroides *sp., *Mobiluncus *sp., Gram-positive cocci, and genital mycoplasma [14].

The association between infections of the upper genital tract and preterm delivery (premature birth) in BV asymptomatic women formed the basis of several trials using antibiotics to prevent preterm delivery in these women; the results of these trials have been contradictory [15-17]. The results of a very large, well-designed trial did not indicate any clear benefit of antibiotic therapy; in addition, the study showed negative results in the subgroup of patients with a history of preterm delivery [18]. Bacterial resistance, timing of the intervention (possibly delayed), and foetal inflammatory syndrome, which can result in early intrauterine death, could help explain such results [19-21].

The study presented here tested an alternative to antibiotics, i.e., so-called probiotics (a type of bacterial therapy), to prevent preterm delivery associated with intrauterine infections originating from bacterial vaginosis. Probiotics have been tested mainly for treating infectious, inflammatory, and allergic conditions that occur in the intestinal, genitourinary, and respiratory tracts [22-24]. Many of the effects of probiotics are not related solely to changes in the microbiota, as indicated by cultivation experiments; rather, many of the beneficial effects of probiotics are related to their immune-modulating effects (i.e., increases in both immunologic and anti-inflammatory activity) [25]. The rationale for the trial presented here was the manipulation of vaginal microbiota to interrupt the infectious/inflammatory process that leads to preterm delivery.

Four small trials have tested the efficacy and safety of selected probiotics (generally, *Lactobacillus rhamnosus *GR-1 associated with *Lactobacillus fermentum *TC-14) for curing urogenital infections, including BV, in asymptomatic non-pregnant women [26-30]. Oral treatments of more than one million of each selected bacillus, administered once or twice daily for 2-8 weeks, showed an efficacy for curing BV (Nugent score) of at least 40%, which was statistically significant. The best results corresponded to the highest dose regimen; most of the treatment effect remained one month after the end of the treatment. No adverse events were reported. However, there is no evidence of the efficacy of probiotics to prevent BV-related conditions, such as preterm delivery and neonatal morbidity.

## 2. General Objectives

To estimate the efficacy of the early administration of specially formulated probiotics to pregnant women with bacterial vaginosis or intermediate-degree infection to prevent the occurrence of spontaneous premature delivery and related neonatal mortality and morbidity.

### 2.1. Specific objectives

a- To assess the presence of bacterial vaginosis and intermediate-degree infections in asymptomatic pregnant women with no risk/indication of elective preterm delivery who were admitted to prenatal care after the 8^th ^and before the 20^th ^week of gestation.

b- To investigate whether the study intervention can reduce the vaginal pH and the Nugent score.

c- To assess the efficacy of an early intervention with special probiotics to treat bacterial vaginosis/intermediate-degree infections and to prevent spontaneous preterm delivery and associated neonatal conditions in positive women, according to item *a*, by conducting a controlled, randomised, patient-allocator-physician/nurse-evaluator-blind trial.

## 3. Methodology

A detailed version of the trial protocol was presented in a previous paper [31].

Asymptomatic pregnant women who were admitted after the 8^th ^and before the 20^th ^week of pregnancy in selected public prenatal services in the city of Rio de Janeiro were evaluated to identify a) excluding clinical conditions associated with elective preterm delivery, symptomatic vaginal conditions, and the recent use of corticotherapy or antibiotic therapy and b) pregnant women with a previous history of preterm delivery.

Gestational age was determined by ultrasound in 71% of the randomised women; in 29% of the randomised women, only the date of the last menstrual period was available upon admission to the trial.

Women who tested positive for syphilis, toxoplasmosis, gonorrhoea, or HIV were excluded. Women reporting vaginal discharge were excluded only if a vaginal smear analysis showed bacterial vaginosis, trichomoniasis, or candidiasis; women with macroscopic genital lesions or microscopic pre-cancerous HPV-related lesions were excluded.

The additional exclusion criteria were applied: multiple gestation, cervical incompetence (cerclage in current gestation), and clinical suspicion of a lower urinary tract infection.

After written informed consent was obtained, a vaginal pH assessment was performed; patients with pH < 4.5 were excluded. A cervicovaginal smear was then obtained to evaluate the presence of BV or an intermediate-degree infection using the Nugent method [32]. Women with Nugent scores < 4 were excluded from the trial.

### Randomisation and Blinding

Randomisation was stratified according to the history of premature delivery (HPD) and blocked.

The sequences for randomisation were electronically generated by an independent research assistant who produced one blocked and randomised list for each stratum using a permuted block design. After written informed consent was obtained, women with Nugent scores > 3 were randomly assigned to receive capsules of either a placebo or probiotics, which were identical in appearance, in sequentially numbered identical containers according to the allocation sequence. Research nurses responsible for the allocation were not directly involved with prenatal care, i.e., with clinical screening. Physicians/nurses and researchers/evaluators were also blinded to the randomisation process.

### Intervention

The trial tested two lactobacilli strains, *Lactobacillus rhamnosus *GR-1 and *Lactobacillus reuteri *RC-14, that were developed and studied by Reid et al. [26-30]. Each capsule contained more than one million bacilli of each strain. The participants were instructed to take two capsules per day up until approximately the 24^th^-26^th ^week of gestation; the treatment duration varied from six to twelve weeks depending on the participant's gestational age at the time of enrolment in the study.

Compliance/adherence, adverse events, and clinical intercurrences were monitored by the research nurse at each routine prenatal visit until the completion of the treatment. Adherence was defined as at least 6 weeks of treatment with ingestion of at least 80% of the prescribed dose. At the post-treatment prenatal care visit, vaginal pH was assessed; a cervicovaginal smear and fluid were obtained to the evaluate changes in these parameters.

The primary outcome was spontaneous preterm delivery between 34 and 37 weeks' gestation. Associated morbidities, early neonatal sepsis, respiratory distress syndrome, bronchopulmonary dysplasia, periventricular leukomalacia, necrotising enterocolitis, and retinopathy of prematurity were the secondary outcomes. The definitions of these conditions were similar to those used by the Vermont-Oxford Network [33].

### Sample size

The estimated prematurity rate for deliveries at < 34 weeks was 6%; the estimated efficacy was 50%. With a 5% significance level, one- and two-sided tests, and 80% power to detect differences in the premature birth rates between the intervention and placebo groups, the trial sample size was estimated to be 1,140 and 1,480, respectively.

## 4. Results

### Flow of patients

**Figure 1:**
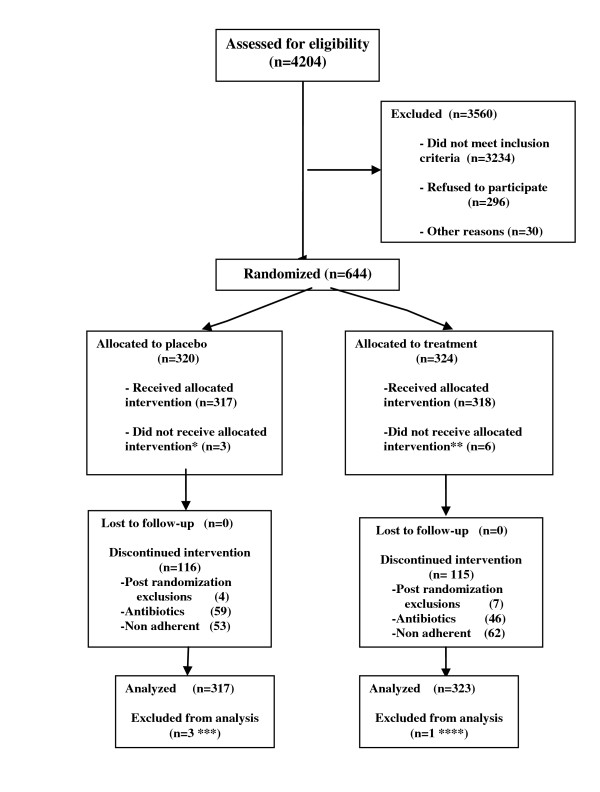
**Observed flow of patients in the trial**. * Returned all capsules (lack of adherence), unknown reason. ** Returned all capsules (unknown reason) (3), family opposed participation in the trial (1), pregnant woman suffered abortion (1), participant presented with UTI (1); the last two events occurred on the day that they were randomly assigned. *** Randomisation error (3): Nugent score < 4 (2); gestational age miscalculation (1). **** Randomisation error (1): Nugent score < 4.

Figure 1 shows the flow of patients according to the protocol and the latest CONSORT guidelines [34-36]. In total, 4,204 pregnant women were screened for the selected clinical conditions; 1,506 were excluded for one or more of the following conditions: hypertension, diabetes, asthma, cervical incompetence, atypical vaginal bleeding, atypical vaginal secretion, HPV, gonorrhoea, syphilis, dysuria, pruritus, burning, corticotherapy, recent antibiotic therapy (within 8 weeks prior to screening), or other miscellaneous clinical factors. Of the remaining patients, 381 pregnant women were not eligible because their gestational age was 20 weeks or greater; 202 were not eligible at the first visit because their gestational age was less than 8 weeks, and they were not available during subsequent prenatal visits; 28 women could not provide the date of their last menstrual period and did not present an ultrasound test; and 233 individuals refused consent for the first phase of the trial.

Vaginal pH was determined for 1,854 women: 546 had pH < 4.5 and were excluded. Of the pH-eligible women, 554 had Nugent scores < 4 and were excluded. Six women refused consent for the intervention phase of the trial. Of the women who were then eligible for randomisation and provided consent, 104 were not randomly assigned because they did not attend the visit or presented with excluding conditions at the prenatal care/randomisation visit (particularly hypertension and symptomatic vaginal infection). Finally, 644 women were randomly assigned, with 324 allocated to the intervention group and 320 placed into the placebo group. Of these women, 9 did not start treatment, 318 initiated treatment with probiotics, and 317 began taking the placebo.

After the initial treatment, 120 women in the placebo group and 122 in the treatment group discontinued the intervention: a) 4 women were excluded from the placebo group (3 women because of pregnancy-induced hypertension and 1 individual was overweight), and 7 were excluded from the treatment group (1 case of umbilical hernia and thrombocytopenia, 4 cases of pregnancy-induced hypertension, 1 case of low platelet count, and 1 instance of a high risk of thrombosis); these patients were referred to high-risk care; b) 59 participants from the placebo group and 46 women from the treatment group required antibiotic therapy and discontinued intervention; and c) 57 women in the placebo group and 69 women in the treatment group were non-adherent. Nearly 62% of the participants finished the trial; none of the randomised participants were lost to follow-up.

### Outcomes

Four patients were excluded from the analysis: 3 from the placebo group (randomisation errors: Nugent score < 4, two patients; gestational age miscalculation, one patient) and 1 from the treatment group (randomisation error: Nugent score < 4).

Only 35 randomly assigned women, or 5%, had a history of premature delivery (HPD). Tables 1 and 2 present the post-randomisation distribution of the relevant variables for the intervention and placebo groups of each stratum of women with and without a history of premature delivery (HPD; NHPD). The distribution of risk variables between the treatment groups of the non-HPD pregnant women was balanced. The distribution of risk variables among the HPD participants was balanced, except for Nugent scores > 8, which were twice as frequent in the intervention group as in the placebo group (ns).

**Table 1:** Post-randomization distribution of relevant risk variables according to treatment group for the stratum of women without history of premature delivery (NHPD)

	**Placebo**			**Treatment**			**Total**		
			
**Factor**	**Frequency**	**%**	**Mean**	**Frequency**	**%**	**Mean**	**Frequency**	**%**	**Mean**
		
Gestacional Age at									
Randomization									
< 12w	9	3.0%	11w.2d	4	1.3%	11w.4d	13	2.1%	11w.2d
(12w-16w)	103	34.1%	14w.2d	115	37.5%	14w.3d	218	35.8%	14w.2d
(16w-20w)	190	62.8%	18w.6d	186	61.2%	18w.3d	376	61.70%	18w.5d
>20w*	2	0.7%	21w.3d	0	0.0%	-	2	0.3%	21w.3d
Total	304	100.0%	17w.1d	305	100.0%	16w.6d	609	100.0%	16w.7d
									
Nugent Score									
<4	2	0.7%	2.5	1	0.3%	1.0	3	0.5%	2.0
4-6	81	26.6%	5.4	90	29.5%	5.4	171	28.1%	5.4
7-8	181	59.5%	7.6	173	56.7%	7.6	354	58.5%	7.6
9-10	40	13.2%	9.2	41	13.4%	9.4	81	13.3%	9.3
Total	304	100.0%	7.2	305	100.0%	7.2	609	100.0%	7.2
									
Lag between elegible									
Nugent score and									
randomization									
< = 2 weeks	180	59.2%	1w.3d	184	60.3%	1w.1d	364	59.8%	1w.2d
>2 weeks	124	40.8%	3w.1d	121	39.7%	3w.3d	245	40.2%	3w.2d
Total	304	100.0%	2w.1d	305	100.0%	2w.0d	609	100.0%	2w.0d
									
pH									
4.5	90	29.6%	4.50	81	26.6%	4.50	171	28.1%	4.50
5.0	151	49.7%	5.00	156	51.1%	5.00	307	50.4%	5.00
5.5 or 6.0	63	20.7%	5.52	68	22.3%	5.54	131	21.5%	5.53
Total	304	100.0%	4.96	305	100.0%	4.99	609	100.0%	4.97
									
Age (years)									
<18	41	13.5%	16.4	39	12.8%	16.4	80	13.1%	16.4
> = 18	263	86.5%	24.8	266	87.2%	24.6	529	86.9%	24.7
Total	304	100.0%	23.7	305	100.0%	23.6	609	100.0%	23.6
									
Height (m)									
< = 1.55	83	27.3%	1.52	72	23.6%	1.52	155	25.5%	1.52
>1.55	221	72.7%	1.63	233	76.4%1	1.63	454	74.5%	1.63
Total	304	100.0%	1.60	305	100.0%	1.60	609	100.0%	1.60
									
									
									
									
Ethnicity									
White	105	34.5%		103	33.8%		208	34.2%	
Black	199	65.5%		200	65.6%		399	65.5%	
NA	0	0%		2	0.7%		2	0.3%	
Total	304	100.0%		305	100.0%		609	100.0%	
									
Nulliparity									
No	109	35.9%		115	37.7%		224	36.8%	
Yes	195	64.1%		190	62.3%		385	63.2%	
Total	304	100.0%		305	100.0%		609	100.0%	
									
Adherence									
No	107	35.2%		104	34.1%		211	34.6%	
Yes	197	64.8%		201	65.9%		398	65.4%	
Total	304	100.0%		305	100.0%		609	100.0%	

*Randomization errors: one error was related to the determination of gestational age and 3 errors were related to the assessment of the Nugent score.

**Table 2:** Post-randomisation distribution of risk variables and adherence according to treatment group for women with a history of premature delivery (HPD)

	**Placebo**			**Treatment**				**Total**		
			
**Factor**	**Frequency**	**%**	**Mean**	**Frequency**	**%**	**Mean**		**Frequency**	**%**	**Mean**
		
Gestacional Age at										
Randomization										
< 12w	2	12.5%	11w.6d	2	10.5%	11w.1d		4	11.4%	11w.4d
(12w-16w)	7	43.8%	14w.3d	7	36.8%	14w.3d		14	40.0%	14w.3d
(16w-20w)	7	43.8%	17w.5d	10	52.6%	19w.4d		17	48.6%	18w.6d
Total	16	100.0%	15w.4d	19	100.0%	16w.5d		35	100.0%	16w.2d
										
Nugent Score										
4-6	5	31.3%	4.8	5	26.3%	5.4		10	28.6%	5.1
7-8	9	56.3%	7.8	9	47.4%	7.7		18	51.4%	7.7
9-10	2	12.5%	9.0	5	26.3%	9.0		7	20.0%	9.0
Total	16	100.0%	7	19	100.0%	7.4		35	100.0%	7.2
										
										
										
Height(m)										
< = 1.55	4	25.0%	1.51	5	26.3%	1.53	9	25.7%	1.52	
>1.55	12	75.0%	1.64	14	73.7%	1.63	26	74.3%	1.63	
Total	16	100.0%	1.61	19	100.0%	1.60	35	100.0%	1.60	
										
Ethnicity										
White	3	18.8%		5	26.3%		8	22.9%		
Black	13	81.2%		14	73.7%		27	77.1%		
Total	16	100.0%		19	100.0%		35	100.0%		
										
Adherence										
No	4	25.0%		7	36.8%		11	31.4%		
Yes	12	75.0%		12	63.2%		24	68.6%		
Total	16	100.05		19	100.0%		35	100.0%		

The outcomes were analysed according to intent-to-treat and actual treatment (explanatory analysis); additional analyses were also performed, and Table 3 provides details on the corresponding denominators, i.e., which participants were included in each analysis. Of 35 HPD women, two participants from the intervention group delivered premature infants (Table 3). For the non-HPD stratum, the following denominators were observed: a) the rates of spontaneous deliveries at < 34 and < 37 weeks of gestation were less than 1% and 2.5%, respectively; no infant was born at less than 30 weeks of gestation; b) the intent-to-treat relative risks of spontaneous premature birth at < 34 and < 37 weeks of gestation were 0.33 (0.03, 3.16) and 0.49 (0.17, 1.44), respectively, which were non-significant (ns) with p = 0.31 and 0.14, (Table 3). Additionally, c) the corresponding actual treatment figures were zero and 0.32 (0.09, 1.19), respectively, which were ns with p = 0.12 and 0.06 (Fisher's exact test, one tail) (Table 3). The intent-to-treat relative risk of spontaneous premature birth at < 37 weeks of gestation for the trial as a whole, including HPD and NHPD participants, was 0.69 (0.26, 1.78), which was ns with p = 0.30, and the corresponding actual treatment figure was 0.54 (0.19, 1.60), which was ns with p = 0.20 (Fisher's exact test, one tail).

**Table 3:** Outcome Analyses

3.1. Premature deliveries related to HPD pregnant women, according to randomization group and adherence							
	**PLACEBO**			**Treatment**			

	**Adherent**	**No-adherent**	**Exclusion (ATB)**	**Adherent**	**No-adherent**	**Exclusion(ATB)**	**Total**

PD<34w.	0	0	0	1	0	0	1
PD<37w.	0	0	0	2	0	0	2
NPD	11	0	5	9	4	4	33
Total	11	0	5	11	4	4	35
PD: Premature Delivery							
NPD:Non-premature delivery							

3.2. Relative risk of premature delivery related to NHPD pregnant women - Intent-to-treat analysis *							

	**Placebo **	**Treatment**	**RR**	**RR 95% CI**	p**		

PD<34w.	3	1	0.330	(0.03,3.16)	0.31		
PD<37w.	10	5	0.495	(0.17,1.43)	0.14		
Total(denominator)*	301	304					
*Randomisation errors were not included.							
							

**3.3. Relative risk of premature delivery related to NHPD pregnant women - Exploratory analysis**							

(actual treatment)*							

	**Placebo**	**Treatment**	**RR**	**RR 95% CI**	p**		

PD<34w.	3	0	0.000	-	0.12		
PD<37w.	9	3	0.326	(0.09, 1.19)	0.06		
Total (denominator)*	188	192					
* Not included: randomization errors, those who did not receive intervention, post- randomisation exclusions, those who discontinued treatment due to antibiotics prescription, and those who were not adherent.							
**Additional Analysis (1)**							
**3.4. Relative risk of premature delivery related to NHPD pregnant women**							

	**Placebo**	**Treatment**	**RR**	**RR 95% CI**	p**		

PD<34w.	3	1	0.333	(0.03, 3.19)	0.31		
PD<37w.	10	5	0.500	(0.17, 1.44)	0.15		
Total (denominator)*	297	297					
*Not included: randomization errors and post-randomisation exclusions							
**Additional Analysis (2)**							
3.5. Relative risk of premature delivery related to NHPD pregnant women*							

	**Placebo**	**Treatment**	**RR**	**RR 95% CI**	p**		

PD<34w.	3	0	0.000		0.12		
PD<37w.	9	3	0.323	(0.09, 1.18)	0.06		
Total (denominator)*	241	249					
							
* Not included: randomisation erros, those who did not receive intervention, post-randomisation exclusions, and those who discontinued treatment due to the prescription of antibiotics.							
** Fisher's exact test, one tail.							

Among the spontaneous preterm babies with < 37 weeks of gestation, targeted neonatal complications occurred in only one infant with < 34 weeks of gestation from the placebo group, who presented with respiratory distress syndrome and suspected early neonatal sepsis.

The recorded adverse events were minor and relatively non-specific; their frequency was low in both trial arms. The frequencies of such events in the intervention and placebo groups was as follows: nausea (5 intervention; 8 placebo), vomiting (6;4), diarrhoea (3;4), sleepiness (0;2), headache (4;1), pyrosis (1;3), stomach ache (2;0), uterine contractions (2;0), nausea/vomiting and headache (1;3 ), vomiting and diarrhoea (1;1), pruritus (0;3), abdominal pain and fainting (1;0), dizziness (1;1), abdominal ache (2;0), abdominal pain and vomiting (1;0), bloody diarrhoea (1;0), and tachycardia (1;1). One maternal death occurred in the placebo group; according to the Committee on Maternal Deaths of the State Health Department, the maternal death was caused by the syndrome of haemolytic anaemia, elevated liver enzymes, and a low platelet count (HELLP syndrome).

## 5. Conclusions

The efficacy of the administration of the tested probiotics at the early second trimester in preventing spontaneous preterm delivery at < 37 weeks of gestation among women without a history of preterm delivery was not determined because the study sample was insufficient to estimate statistically significant intent-to-treat effects; additional studies are necessary to evaluate this intervention among these women.

## 6. Discussion

The findings of this trial lacked a sufficient sample size, as indicated by the *p *values, to permit reliable inferences concerning the efficacy of the tested probiotics in preventing spontaneous premature births. However, all point estimates of relative risks for the non-HPD stratum, and the actual treatment analyses in particular, were less than 0.5.

Although the number of screened women was higher than estimated, the planned sample size, based on available international and national/local information, was not attained because the attrition rates were high. The trial had to be interrupted mainly because of a lack of on-going financial support. The main sources of attrition were the higher-than-expected prevalence of clinical, vaginal pH, and Nugent exclusion conditions. In addition, the intent-to-treat effect was likely diminished because a significant proportion of the randomised women exhibited conditions that required antibiotic therapy after the initiation of treatment (such as symptomatic vaginal discharge and urinary tract infection); this problem was more frequent in the placebo group and led to a discontinuation of the treatment. The intent-to-treat effect was also affected by a higher-than-expected lack of adherence, which was more frequent in the intervention group. Several randomly assigned women were excluded and discontinued treatment because they suffered abortion or developed conditions for exclusion that were strongly associated with the indication of preterm delivery, such as hypertension, and were referred to high-risk care (Figure 1). However, these women were considered in the intent-to-treat analyses.

The participants were instructed to take the treatment/placebo until approximately the 24^th^-26^th ^week of gestation. By this time, at the end of the second trimester, BV is less likely to occur and commonly remits; however, the main concern was to avoid delivery at < 34 weeks, and an eventual re-infection and progression would take approximately 8 weeks to occur [9].

Three out of five women who spontaneously delivered prior to the 34^th ^week of gestation exhibited initial Nugent scores of 9 or 10; these scores were present in less than 15% of the total number randomised women, which is consistent with the hypothesis that BV is a risk factor for premature birth in the study population [8,37-39].

Four out of five spontaneous deliveries at < 34 weeks of gestation were initiated with the preterm premature rupture of membranes (PPROM); only one woman was admitted for premature labour without PPROM. These 5 cases could be associated with intrauterine infection, including BV [20,40-43]. The non-PPROM case was not associated with any recorded maternal or foetal condition, other than BV, that can cause PD. Two cases of PPROM were associated with oligohydramnios; such an association has been shown to be related to infection. One case of PPROM occurred in a woman with HPD, and the remaining case of PPROM was not associated with any recorded maternal or foetal condition, other than BV, that could cause PPROM and PD [43].

Because HPD is considered to be a major predictor of subsequent PD, including very premature births [43], women were stratified for HPD and randomly assigned with a stratum-specific randomisation list. The number of HPD women enrolled in the trial was relatively small (35 cases); the corresponding intervention arm received more severe cases of BV than the placebo arm (Table 2). Table 3a, which shows the related outcomes, has two blank cells that indicate missing data, which prevents any meaningful analysis. Furthermore, the fact that such HPD cases are generally considered especially difficult to understand and solve [7,42] prompted the authors to analyse the data from the non-HPD group separately.

The study protocol used ultrasound as the standard method for determining gestational age. Two premature infants had ultrasound-determined gestational ages of 30 6/7 and 32 4/7 weeks, which were different from the gestational ages estimated by the attending neonatologists (33 6/7 and 30 6/7 weeks, respectively). However, none of the premature infants were born before the 30^th ^week, which is consistent with the relatively low rate of observed morbid events and the birth weights in the study.

Neonatal records were reviewed by two research neonatologists. All 5 infants spontaneously delivered at less than 34 weeks of gestation were discharged from the hospital. Of these five cases, only one newborn was diagnosed with any of the morbid conditions under study; that infant, whose mother belonged to the placebo group and non-HPD stratum, presented with pneumonia as well as possible respiratory distress syndrome and sepsis (Table 3). Pneumonia was diagnosed based on the presence of risk factors for infection, clinical signs, and radiographic findings. Given that radiographic findings in pneumonia can be identical to those of hyaline membrane disease [44], it was not possible to dismiss HMD in the immediate postpartum period in this case. Sepsis was suspected because of an immature-to-total neutrophil ratio higher than 0.2, although a blood culture was not positive, as required by the Vermont Oxford Network criteria. Other sources consider an immature-to-total neutrophil ratio higher than 0.2 to be an equally or more accurate criterion for early sepsis compared with a positive blood culture result [44]. The infant progressed well and did not develop bronchopulmonary dysplasia.

Interestingly, the case with delivery at < 34 weeks of gestation, in which the Nugent score decreased from 9 to 2 by the end of the trial (normal vaginal mucosa), was an adherent woman with history of preterm delivery from the intervention arm who initiated treatment after the 20^th ^week of pregnancy. It has been argued that HPD is caused by chronic intrauterine infection [45]. One explanation is that lactobacilli that colonise the vagina of such women, and of women with repeated BV, produce an insufficient amount of lactic acid and other bactericidal substances [46,47]. In addition, the gestational age at which the vaginal infection occurs and treatment/intervention begins may interact with the maternal and foetal immune-genetic profile/response to result in birth at less than 34 weeks of gestation [48-51]. After the 20^th ^week of gestation, the infection may have progressed to the extent that an undesired outcome could be determined [5,19].

To better understand the significance of the outcomes in the present trial, the early (early mid-trimester) and later (late mid-trimester) cervical immune-genetic profiles (i.e., selected cytokines and related genetic polymorphisms) of the randomised women are currently being analysed and may be helpful in dismissing or reinforcing the biological plausibility and, therefore, the prospects for a positive role of probiotics in the prevention of spontaneous premature births associated with bacterial vaginosis.

### Ethical issues

The trial was approved by an Institutional Review Board and by the National Review Board (CONEP). The trial was monitored by an independent data monitoring committee, which performed two interim analyses of data related to the main (PD) and secondary outcomes (April 2007 and July 2008), including possible adverse events; the committee received special assistance for unblinding the group allocations. The committee did not recommend the interruption of the trial. The trial was conducted according to ICH/GCP regulations and local regulations for clinical trials.

### Registration

The study was registered at the NIH register platform with identifier NCT00303082.

Sponsors: The trial was supported by grants from FIOCRUZ/Brazilian Health Ministry, SAS/Brazilian Health Ministry, and the State of Rio de Janeiro Research Foundation.

## List of Abbreviations

BV: bacterial vaginosis; HPD: history of premature delivery,PD: premature delivery; PPROM: preterm premature rupture of membranes,RR: relative risk.

## Competing interests

The authors declare that they have no competing interests.

## Authors' contributions

LKS and MEML contributed to the original conception of the trial. MR and MELM participated in the definition of neonatal outcomes and in the design of the neonatal data collection process. LKS, AB, FG, AAH, MRB, KGC, and MBA contributed to the design of the prenatal phase and laboratory procedures of the trial and specified most of the operational procedures. LKS and MBA estimated the study sample and participated in the information system design and the elaboration and execution of the data analysis. All of the authors contributed to the operational phases of the trial. All of the authors participated in drafting the manuscript and gave final approval of this version for publication.
